# Inhibition of Intestinal Adenoma Formation in *APC^Min/^* ^+^ Mice by Riccardin D, a Natural Product Derived from Liverwort Plant *Dumortiera hirsuta*

**DOI:** 10.1371/journal.pone.0033243

**Published:** 2012-03-14

**Authors:** Hui-Ping Liu, Zu-Hua Gao, Shu-Xiang Cui, De-Fu Sun, Yan Wang, Cui-Rong Zhao, Hong-Xiang Lou, Xian-Jun Qu

**Affiliations:** 1 Department of Pharmacology, School of Pharmaceutical Sciences, Shandong University, Jinan, China; 2 Department of Pathology and Laboratory Medicine, University of Calgary and Calgary Laboratory Services, Calgary, Alberta, Canada; 3 Department of Pharmacology, Institute of Materia Medica, Shandong Academy of Medical Sciences, Jinan, China; Faculty of Pharmacy, Ain Shams University, Egypt

## Abstract

**Background:**

Mutation of tumor suppressor gene, adenomatous polyposis coli (*APC*), is the primary molecular event in the development of most intestinal carcinomas. Animal model with *APC* gene mutation is an effective tool for study of preventive approaches against intestinal carcinomas. We aimed to evaluate the effect of Riccardin D, a macrocyclic bisbibenzyl compound, as a chemopreventive agent against intestinal adenoma formation in *APC^Min/+^* mice.

**Methods:**

*APC^Min/+^* mice were given Riccardin D by p.o. gavage for 7 weeks. Mice were sacrificed, and the number, size and histopathology of intestinal polyps were examined under a microscope. We performed immunohistochemical staining, western blotting, reverse transcriptase-polymerase chain reaction (RT-PCR) and enzyme-linked immunosorbent assay (ELISA) in intestinal polyps to investigate the mechanism of chemopreventive effect of Riccardin D.

**Results:**

Riccardin D treatment resulted in a significant inhibition of intestinal adenoma formation, showing a reduction of polyp number by 41.7%, 31.1% and 44.4%, respectively, in proximal, middle and distal portions of small intestine. The activity of Riccardin D against polyp formation was more profound in colon, wherein Riccardin D decreased polyp number by 79.3%. Size distribution analysis revealed a significant reduction in large-size polyps (2–3 mm) by 40.0%, 42.5% and 33.3%, respectively, in proximal, middle and distal portions of small intestine, and 77.8% in colon. Histopathological analysis of the intestinal polyps revealed mostly hyperplastic morphology without obvious dysplasia in Riccardin D-treated mice. Molecular analyses of the polyps suggested that the inhibitory effect of Riccardin D on intestinal adenoma formation was associated with its abilities of reduction in cell proliferation, induction of apoptosis, antiangiogenesis, inhibition of the Wnt signaling pathway and suppression of inflammatory mediators in polyps.

**Conclusions:**

Our results suggested that Riccardin D exerts its chemopreventive effect against intestinal adenoma formation through multiple mechanisms including anti-proliferative, apoptotic, anti-angiogenic and anti-inflammatory activity.

## Introduction

Colorectal cancer (CRC) is the second leading cause of cancer morbidity and mortality worldwide. Most tumors arise sporadically (90%) and heritable cases constitute only 5 to 10% of all CRC population [1]–[3]. The mutation of tumor suppressor gene, adenomatous polyposis coli (*APC*), was found in all CRCs with familial adenomatous polyposis (FAP), and in approximately 80% of sporadic CRCs [4]–[6]. The normal function of *APC* protein is to degrade β-catenin through the Wnt signaling transduction pathway [6]. Dysregulation of the Wnt signaling pathway from *APC* gene mutation results in increase of β-catenin expression in nucleus. In the nucleus, the transcription factor, T cell factor/lymphoid enhancer factor (TCF/LEF) will be transactivated by β-catenin leading to an increased expression of genes that regulate cell proliferation and apoptosis such as cyclin-D1 and c-Myc. The *APC* gene mutation mouse (*APC^Min/+^*) has an autosomal dominant heterozygous nonsense mutation of the mouse *APC* gene at codon 850, homologous to the human germ line and somatic *APC* mutations [7]. Thus, *APC^Min/+^* mouse has been well recognized as the standard experimental model for the study of intestinal carcinogenesis because it allows the tumors to develop spontaneously in the intestinal tract. This model is particularly advantageous for testing chemopreventive agents targeted against early-stage tumorigenesis because scores of adenomas grow to a grossly detectable size within a few months [8].

**Figure 1:**
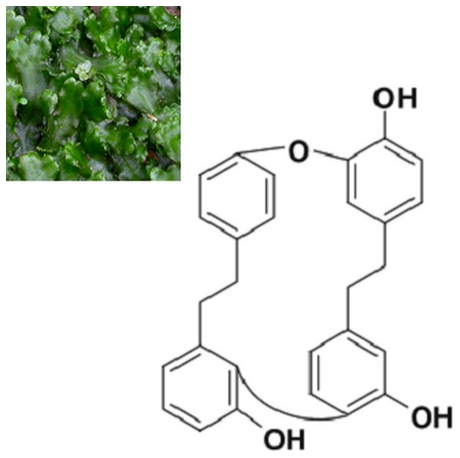
The liverwort plant *Dumortiera hirsuta* and chemical structure of Riccardin D.

Macrocyclic bisbibenzyls are a special class of liverwort-derived components that belong to the family of phenolic compounds. Riccardin D, a macrocyclic bisbibenzyl, was isolated from the liverwort plant *Dumortiera hirsute* (Fig. 1) [9]. Our previous studies showed that Riccardin D could interfere with the formation of biofilm in Candida albicans [10]. Recently, Riccardin D was found to inhibit the proliferation of human leukemia cell lines including HL60, K562 and its multidrug resistant (MDR) counterpart K562/A02 cells. Riccardin D was shown to induce apoptosis of leukemia cells through targeting DNA topoisomerase II [11]. Riccardin D inhibited tumor angiogenesis in human lung carcinoma H460 xenografts in mice without apparent toxicity to animals [12]. Thus, Riccardin D may have a chemotherapeutic and possibly chemopreventive effect on cancers. In this study, we first evaluated the chemopreventive effects of Riccardin D on spontaneous intestinal adenoma formation in *APC^Min/+^* mice. We then investigated the molecular mechanism of Riccardin D on the inhibition of intestinal adenoma formation. Our results provide scientific evidence that supports Riccardin D as a potential chemopreventive regimen for intestinal cancers derived from *APC* gene mutation.

## Materials and Methods

### Drug

Riccardin D was isolated from the liverwort plant *Dumortiera hirsuta* by our group and its structure was identified as reported previously (Fig. 1) [9], [11]. The purity of Riccardin D as measured by high performance liquid chromatography (HPLC) was 98.6%.

### Animal model and drug treatment protocol

Male *APC^Min/+^* mice obtained from The Jackson Laboratory (Bar Harbor, USA) were crossed with wild-type C57BL/6 female mice to generate *APC^Min/+^* mice [6], [7], [13]. A total of 20 female *APC^Min/+^* mice (age, 4 wk) were randomly divided into two groups. After one week acclimation, the two groups of 10 mice (5 wk) each were given the control (5% amylum) and Riccardin D 80 mg/kg by p.o. gavage daily (0.2 ml/10 g body weight) for 7 consecutive weeks. Selection of Riccardin D dose was based on our previous studies [12], [14]. Animals were weighed weekly and checked daily for any signs of illness. The research protocol was approved strictly in accordance with the institutional guidelines of Animal Care and Use Committee at Shandong University. The permit number was SYXK(LU)20100418.

### Quantification of macroscopic and microscopic intestinal adenomas

Following sacrifice, small intestine and colon were removed, sliced longitudinally, rinsed with saline and spread onto microscope slides. Small intestine was divided by length into three equal sections (proximal, middle, and distal segments) according to previous reports [15]–[17]. Polyps on intestinal segments were counted, and their sizes were measured with digital caliper under a dissecting microscope. The intestines were subsequently embedded in paraffin and stained with hematoxylin and eosin (H&E) for microscopic examination.

### Immunohistochemistry staining and quantification

Sections of 4 µm thickness were cut from formalin-fixed intestinal polyps. After deparaffinization, antigen retrieval, endogenous peroxidase activity was blocked by incubation with 3% hydrogen peroxide in methanol for 10 min. The sections were then washed twice in phosphate-buffered saline (PBS, pH 7.4) for 5 min. Non-specific binding was blocked by incubation with 5% bovine serum albumin for 20 min. After incubation with primary antibodies, the sections were washed and treated with biotinylated anti-immunoglobulin, washed, reacted with avidin-conjugated horseradish peroxidase H complex, and incubated in diaminobenzidine and hydrogen peroxide. The sections were rinsed in distilled water, counterstained with hematoxylin, and mounted [18]. The primary antibodies included anti-proliferating cell nuclear antigen (PCNA) (2586, 1∶100 dilution), anti-β-catenin (9562, 1∶100 dilution), anti-cyclin D1 (2922, 1∶100 dilution), anti-cyclooxygenase-2 (COX-2) (4842, 1∶50 dilution, Cell Signaling) and anti-vascular endothelial growth factor (VEGF) (sc7269, 1∶100 dilution, Santa Cruz). For the subsequent reaction, SABC kit (Bostar, China) was used according to manufacturer's instruction. Nuclear positivity of PCNA and cyclin D1 were quantified as percentage of positive cells per analyzed area as described previously [15]. The cytoplasmic and nuclear staining of β-catenin, and cell membrane staining of COX-2 and VEGF were quantified by scoring the intensity as 0 (no staining), +1 (very weak), +2 (weak), +3 (moderate), and +4 (strong) at five randomly selected fields at 400× magnification in each sample [15], [16].

Apoptotic cells in intestinal polyps were identified by terminal deoxynucleotidyl transferase-mediated dUTP nick end labeling (TUNEL) staining using *In Situ* Cell Death Detection Kit (Roche, Germany) [19]. Serial 4-µm sections were cut from formalin-fixed intestinal polyps. The staining was performed according to manufacturer's instruction. The proportion of the TUNEL-positive cells in at least three mice in one group was scored in randomly chosen fields under a microscope [20].

CD34 immunohistochemical staining was performed to examine the angiogenesis in intestinal polyps. Sections of polyps between 2–3 mm were used for analysis of angiogenesis. After incubation with anti-CD34 (BA0532, Boster, China) at 4°C, the sections were treated with biotinylated anti-immunoglobulin, washed, reacted with avidin-conjugated horseradish peroxidase H complex, and then incubated in diaminobenzidine and hydrogen peroxide. The slides were rinsed in distilled water, counterstained with hematoxylin, and mounted [21]. For angiogenesis analysis, all morphological structures with a lumen surrounded by CD34-positive endothelial cells were considered as blood microvessels. Microvascular density (MVD) was calculated by counting CD34 positive vessels as described previously [22].

### Western blotting analysis

Western blotting analysis was performed to evaluate the expressions of cancer growth-related proteins in intestinal polyps. Polyps were incubated with 50 µl RIPA lysis buffer at 4°C for 30 min and the lysates (30 µg of protein per lane) were fractionated by SDS-PAGE. The proteins were electro-transferred onto PVDF membranes and then the expressions were detected using dilutions of the primary antibodies. The primary antibodies included anti-NF-κB (sc-8008), anti-p-NF-κB Ser^536^ (sc-33020), anti-FGF-2 (sc79, Santa Cruz); anti-caspase-3 (9662), anti-caspase-9 (9502), anti-cleaved PARP (9541), anti-Bcl-2 (2872), anti-Bax (2772, Cell Signaling); anti-TNF-α (BA0131, Boster, China) and anti-β-actin (ab6276, Abcam). The PVDF membranes were washed in 0.05% Tween-20/TBS and then incubated with horseradish peroxidase-conjugated secondary antibody. The bound antibodies were visualized using an enhanced chemiluminescence reagent (Millipore, USA) and quantified by densitometry using ChemiDoc XRS+ image analyzer (Bio-Rad, USA). Densitometric analyses of bands were adjusted with β-actin as loading control [23]. Triplicate experiments with triplicate samples were performed.

### Reverse transcription-polymerase chain reaction

Reverse transcriptase-polymerase chain reaction (RT-PCR) assay was used to analyze the expressions of COX-2 and TNF-α in intestinal polyps [24]. Polyps were collected and total RNA was extracted using the RNAeasy kit according to manufacturer's instruction (Sangon, China). RNA quality was confirmed by the ratio of A260/A280 (1.8–2). The concentration of total RNA was measured by detecting absorbance at 260 nm (A260). Then, reverse transcription (RT) was carried out with 0.1 µg extracted RNA using First Strand cDNA Synthesis Kit (Toyobo, Japan). The following primers (Genecore Biotech, China) were used for the specific amplification of COX-2: forward, 5′-CCAGATGCTATCTTTGGGGA-3′, reverse, 5′-GCTCGGCTTCCAGTATTGAG-3′; TNF-α: forward, 5′-TGCCTATGTCTCAGCCTCTTC-3′, reverse, 5′-GAGGCCATTTGGGAACTTCT-3′). The expressions of β-actin (forward, 5′-GACTACCTCATGAAGATCCT-3′, reverse, 5′-CCACATCTGCTGGAAGGTGG-3′) were used as an internal control. The reaction was performed at 37°C for 15 min, 98°C for 5 min; and 35 cycles at 94°C for 30 s, 55.5°C for 30 s and 68°C for 1 min. PCR products were specified on 2.5% agarose gels containing 0.5 µg/ml of ethidium bromide and photographed under a UV transilluminator. AlphaEaseFC software was used to analyze the relative light intensities. Triplicate experiments with triplicate samples were performed.

### Assay for prostaglandin E_2_ levels

Small intestinal polyps and normal mucosa (100 mg) were homogenized in 1 ml homogenization buffer (0.1 mol/L phosphate buffer, pH 7.4 containing 1 mmol/L EDTA and 10 µmol/L indomethacin) with polytron-type homogenizer. The homogenates were centrifuged at 14,000 g for 10 min at 4°C, and then the supernatants were aliquoted and stored at −80°C for analysis. The levels of prostaglandin E_2_ (PGE_2_) were measured using ELISA kit (Cayman Chemical, USA) according to manufacturer's protocol [15].

### Statistical analysis

Data were described as Mean ± S.D. Comparison between *APC^Min/+^* control and Riccardin D treatment group were conducted by two-tailed Student's *t* test using the SPSS/Win13.0 software (SPSS, Inc., Chicago, Illinois). P value less than 0.05 was considered statistically significant.

## Results

### General observation

Riccardin D treatment was generally well tolerated by mice during the long-term treatment. There is no apparent difference between the treated and control mice in their body weight, the function of liver and kidney, and peripheral blood elements count (data not shown).

### Riccardin D prevents intestinal adenoma formation in APC^Min/+^ mice

**Figure 2:**
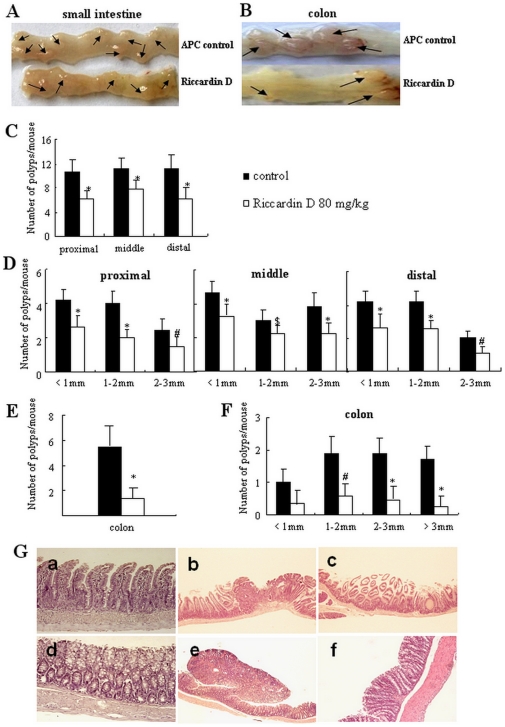
Riccardin D prevented spontaneous intestinal polyposis in *APC^Min/+^* mice. (A and B): Representative pictures of distal small intestinal and colon polyps; (C): The number of polyps per mouse in different parts of small intestines; (D): The size distribution of polyps in different part of small intestines; (E): The number of polyps per mouse in colons; (F): The size distribution of polyps in colons. *, p<0.001; #, p<0.01; $, p<0.05 *versus* control. (G): Histological analysis of intestinal polyps. Sections of small intestine and colon from control or Riccardin D-treated mice were stained with hematoxylin and eosin (H&E) and analyzed under microscopy. **a**, Normal histology of small intestinal mucosa (100×). **b**, Small intestine of control mice showed an area of villus effacement and formation of a polyp with dysplastic glands. The central part of the polyp showed surface erosion and necrosis (40×). **c**, Riccardin D-treated small intestine showed an area of epithelial hyperplasia without obvious dysplasia. The villus architecture in polypoid area is well preserved (40×). **d**, Normal histology of colonic mucosa (100×). **e**, Colon from control mice showed a large pedunculated tubular adenoma (40×). **f**, Colon in mucosa of Riccardin D-treated mice showed well preserved crypt architecture with normal surface maturation. The surface epithelium showed minimal evidence of hyperplasia (40×).

In *APC^Min/+^* mice, all polyps on intestines were histologically identified as adenomas. Riccardin D treatment resulted in a strong inhibition of intestinal adenoma formation in terms of decreased polyp number, size, and appearance in small intestine and colon (Fig. 2A, B). At age of 12 weeks, mice in control group developed 10.8, 11.3, and 11.2 polyps on average in proximal, middle, and distal portions of small intestine, respectively. As shown in Fig. 2C, the number of polyps in Riccardin D-treated mice were decreased by 41.7% (p<0.001), 31.1% (p<0.001), and 44.4% (p<0.001), respectively (Table 1). Size distribution analysis of polyps in small intestine showed differential Riccardin D efficacy depending on intestinal segment and polyp size (Table 2). Riccardin D reduced number of <1 mm size polyps by 35.3% (p<0.001) in proximal, 31.6% (p<0.001) in middle, and 37.3% (p<0.001) in distal segments; 1 to 2 mm size polyps by 50.0% (p<0.001) in proximal, 25.0% (p = 0.015) in middle, and 41.2% (p<0.001) in distal segments ; and 2 to 3 mm size polyps by 40.0% (p = 0.004) in proximal, 42.5% (p<0.001) in middle, and 33.3% (p = 0.005) in distal segments (Fig. 2D). Bigger-size polyps (>3 mm) were not observed in small intestines (Table 2).

**Table 1:** The number of polyps per mouse in different parts of small intestine and colon.

	Polyp number per mouse			
	Proximal^a^	Middle^a^	Distal^a^	Colon
Control	10.8±1.7	11.3±1.6	11.2±2.2	6.2±2.8
Riccardin D	6.2±1.3	7.6±1.5	6.3±1.8	1.2±0.9
Inhibition (%)	41.7*	31.1*	44.4*	80.6*

a , different parts of small intestine;

* , p<0.001 *versus* control.

**Table 2:** The size distribution of polyps per mouse in different part of small intestine and colon.

Polyp size	Proximal^a^		Middle^a^		Distal^a^		Colon	
(mm)	Control	Riccardin D	Control	Riccardin D	Control	Riccardin D	Control	Riccardin D
<1.0	4.2±0.6	2.6±0.7	4.6±0.7	3.3±0.8	4.2±0.6	2.6±1.0	1.0±0.9	0.3±0.5
1.0–2.0	4.0±0.8	2.0±0.5	3.0±0.7	2.3±0.5	4.2±0.6	2.5±0.5	1.8±1.2	0.4±0.5
2.0–3.0	2.4±0.7	1.5±0.5	3.7±0.8	2.2±0.6	1.8±0.4	1.2±0.4	1.8±0.8	0.4±0.5
>3.0	-	-	-	-	-	-	1.2±0.4	0.2±0.4

a , different parts of small intestine.

Most prominent Riccardin D effect was observed on inhibition of polyp number and size in colon. Riccardin D strongly decreased number of colonic polyps by 80.6% (p<0.001, Fig. 2E, Table 1). Size distribution analysis of colonic polyps showed a strong decrease in <1 mm (66.7%, p = 0.051), 1 to 2 mm (70.8%, p = 0.004), 2 to 3 mm (77.8%, p<0.001) and in >3 mm (85.3%, p<0.001) size polyps in Riccardin D-treated mice compared with control (Fig. 2F).

Histological analysis of the intestinal polyps in control mice revealed well formed tubular adenomatous polyps with low grade dysplasia and focal high grade dysplasia in some large polyps (Fig. 2G-b, 2G-c). In Riccardin D-treated mice, the polypoid area in mucosa of intestines mostly showed hyperplastic morphology without obvious dysplasia (Fig. 2G-e, 2G-f).

### Decrease of β-catenin and cyclin D1 expression in intestinal polyps

**Figure 3:**
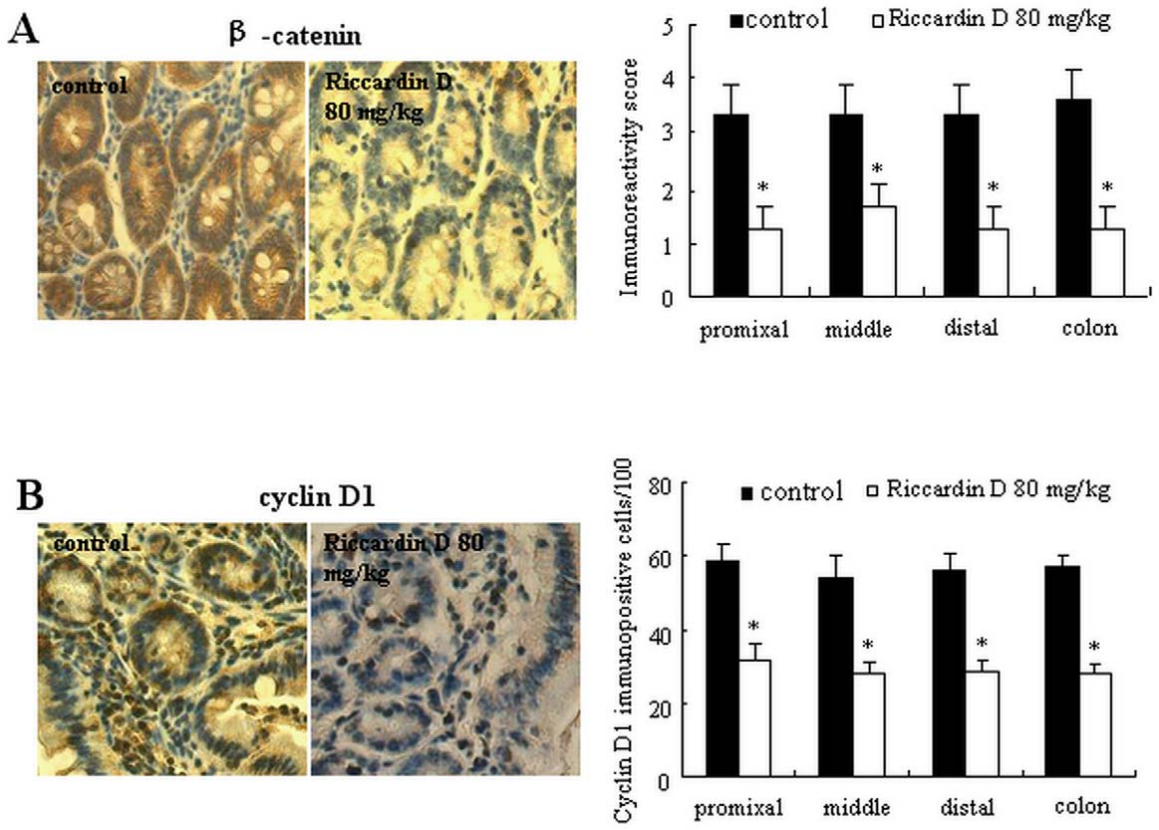
Riccardin D decreased expression of β-catenin and cyclin D1 in intestinal polyps. Intestinal sections were processed for β-catenin and cyclin D1 immunohistochemistry staining. Intestinal sections of control and Riccardin D-treated mice showed brown-colored PCNA-positive (A) and cyclin D1-positive cells (B) in polyps on small intestines and colons (400×). Bar represents the mean ± S.D of six animals. *, p<0.001 *versus* control.

To investigate whether Riccardin D affected the Wnt signaling pathway, we examined two of its main downstream targets, β-catenin and cyclin D1. As shown in Fig. 3, Riccardin D treatment resulted in a strong decrease of these molecules. Quantification of the immunohistochemistry staining indicated the decrease of nuclear β-catenin positivity by 66.7% (p<0.001), 60.0% (p<0.001), and 61.9% (p<0.001; Fig. 3A) and cyclin D1-positive cells by 46.7% (p<0.001), 54.3% (p<0.001) and 51.4% (p<0.001; Fig. 3B) in polyps from proximal, middle, and distal portions of small intestine, respectively, and in colon the inhibition rates of β-catenin and cyclin D1 were 68.2% (p<0.001) and 50.7% (p<0.001; Fig. 3A and B), respectively.

### Suppression of NF-κB and its related inflammation factors

**Figure 4:**
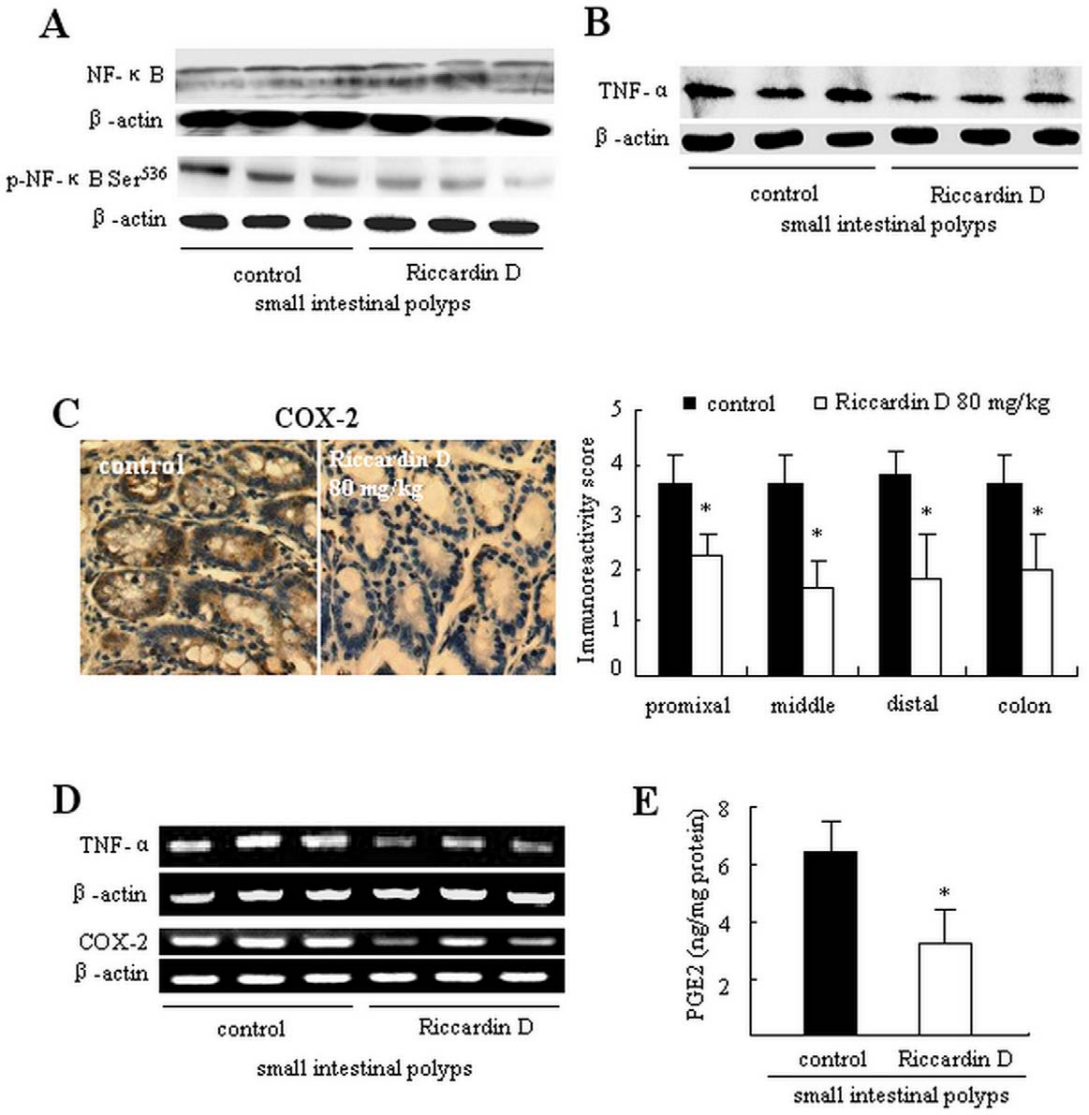
Riccardin D suppressed inflammation by decreasing COX-2 and NF-κB in intestinal polyps. (A): The inhibition of NF-κB and p-NF-κB Ser^536^ in intestinal polyps of Riccardin D-treated mice (n = 3). (B): The inhibition of TNF-α in intestinal polyps of Riccardin D-treated mice (n = 3). (C): The inhibition of COX-2 expression in polyps on small intestines and colons of Riccardin D-treated mice. The bars indicate mean ± S.D. (n = 6). (D): The decrease of COX-2 mRNA in polyps on small intestines as estimated by RT-PCR analysis (n = 3). (E): The content of PGE_2_ in small intestinal polyps was decreased in Riccardin D-treated mice as measured by ELISA analysis. The bars indicate mean ± S.D. (n = 3). *, p<0.001 *versus* control.

Western blotting analysis showed that the expression and activity of NF-kB were highly elevated in intestinal polyps of *APC^Min/+^* mice. Riccardin D significantly decreased NF-kB expression. As shown in Fig. 4A, the level of total NF-κB p65 protein in small intestinal polyps was strongly decreased by 66.2% (p<0.001) of control. Further analysis of p-NF-κB Ser^536^, the active form of NF-κB, was markedly decreased by 73.2% (p<0.001, Fig. 4A, bottom) of control.

We then examined the inhibitory effect of Riccardin D on the expressions of TNF-α, COX-2 and PGE_2_ in intestinal polyps. As shown in Fig. 4B, the level of TNF-α in intestinal polyp was significantly decreased by Riccardin D as detected by western blotting assay. Fig. 4C showed a strong COX-2 immunoreactivity in intestinal polyps, which was markedly decreased by Riccardin D. Quantification of the immunostained cells showed that Riccardin D significantly decreased COX-2 immunoreactivity by 38.9% (p<0.001) in proximal, 55.6% (p<0.001) in middle, 52.6% (p<0.001) in distal, and 44.4% (p<0.001) in polyps of small intestine and colon (Fig. 4C, right).

The inhibitory effect of Riccardin D on TNF-α and COX-2 expression was further evidenced by RT-PCR analysis of small intestinal polyps. As shown in Fig. 4D, the mRNA levels of TNF-α and COX-2 were significantly decreased by 35.4% (p<0.01) and 54.5% (p<0.001) of control, respectively.

We measured the content of PGE_2_, which is an important downstream product of COX-2, in intestinal polyps by ELISA analysis. PGE_2_ levels in small intestinal polyps were elevated (3.5-fold) compared with those in wild-type small intestinal tissue (data not shown). Riccardin D markedly decreased PGE_2_ by 50.0% (p<0.001; Fig. 4E), implying that the activity of COX-2 was suppressed.

### Riccardin D prevents proliferation of intestinal polyps and triggers apoptosis via a caspase-dependent pathway

**Figure 5:**
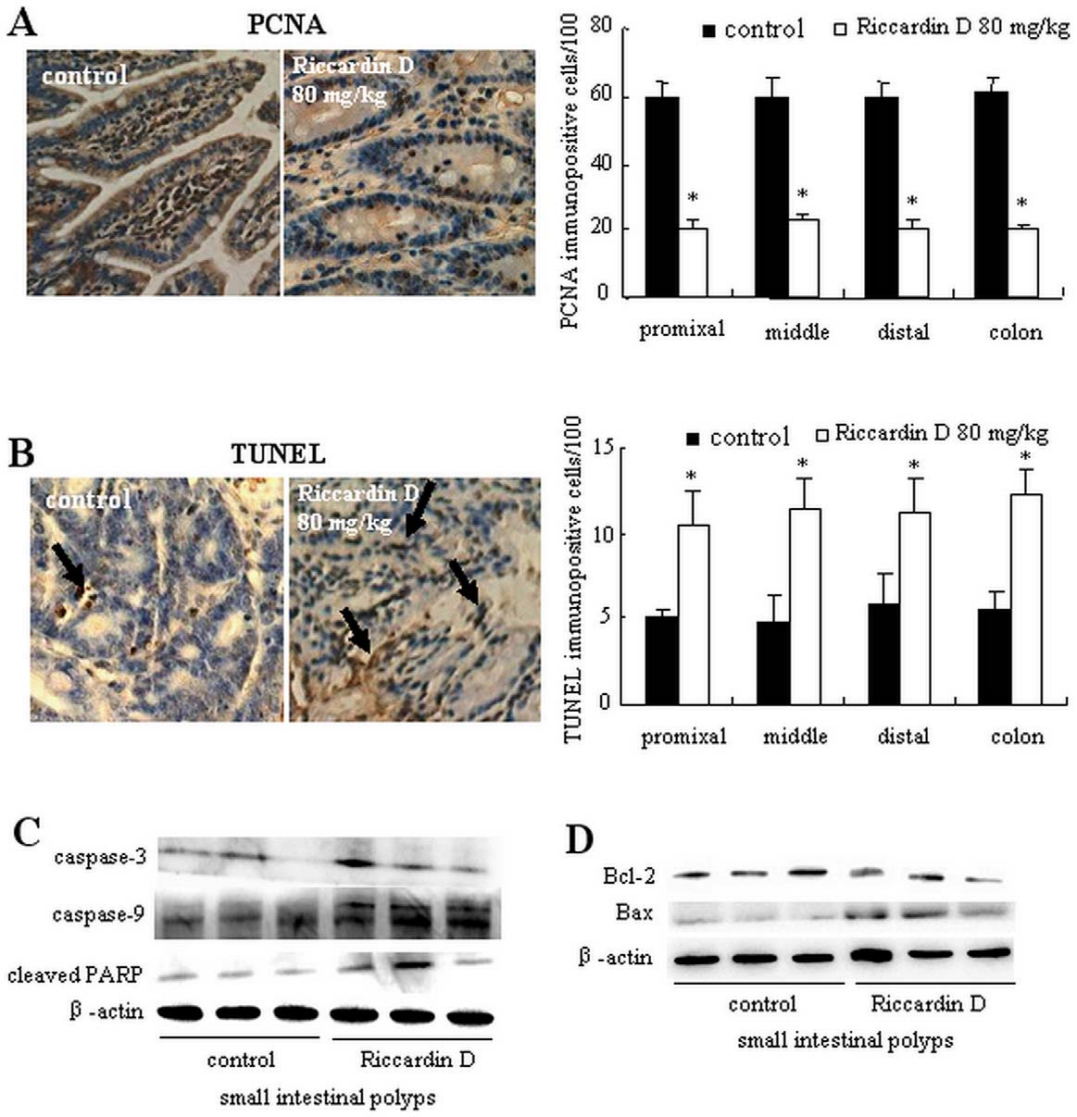
Riccardin D decreased proliferation and induced apoptosis in intestinal polyps. (A and B): Intestinal polyps of control and Riccardin D-treated mice showed brown-colored PCNA-positive (A) and TUNEL-positive (B) cells in polyps (400×). Quantitative data for proliferation and apoptosis were determined as number of PCNA-positive, TUNEL-positive cells×100/total number of cells, respectively. The bars represent mean ± S.D. of six animals. *, p<0.001 *versus* control. (C and D): The alteration of apoptotic proteins in polyps on small intestine as estimated by western blotting analysis. (C): The levels of caspase-9, caspase-3 and cleaved PARP in intestinal polyps were increased by Riccardin D (n = 3). (D): Bcl-2 was decreased and Bax was increased in intestinal polyps in Riccardin D-treated mice (n = 3).

To assess whether Riccardin D efficacy is associated with its anti-proliferative and pro-apoptotic effects, we examined the expressions of PCNA, TUNEL and apoptotic proteins in intestinal polyps. Microscopic examination of tissue sections showed a decrease in PCNA (Fig. 5A) and an increase in TUNEL (Fig. 5B) in polyps from Riccardin D-treated mice compared with those from control mice. Quantification of PCNA staining showed 65.5% (p<0.001), 62.2% (p<0.001), 65.1% (p<0.001) and 67.0% (p<0.001) reduction in proximal, middle, distal portions of small intestine and colon polyps by Riccardin D. Quantitative data showed that Riccardin D treatment resulted in increases of TUNEL-positive cells in intestinal polyps by 51.9% (p<0.001) in proximal, 57.9% (p<0.001) in middle, and 48.2% (p<0.001) in distal portions of small intestine and 54.1% (p<0.001) in polyps of colon (Fig. 5B).

Western blotting analysis indicated that Riccardin D could activate the caspase cascade pathway as demonstrated by increases of caspase-9, caspase-3 and cleaved PARP. As shown in Fig. 5C, the levels of caspase-3, caspase-9 and cleaved PARP in small intestinal polyps were significantly increased in Riccardin D-treated mice. The increases of caspase-3, caspase-9 and cleaved PARP were 75.0% (p<0.001), 70.9% (p<0.001), and 50.9% (p<0.001) of control, respectively. Further analysis of proapoptotic protein Bax and antiapoptotic protein Bcl-2 showed strong increases of Bax activation and decreases of Bcl-2 expression (Fig. 5D). These results indicated that the induction of apoptosis by Riccardin D might be involved in the inhibition of intestinal adenoma formation in *APC^Min/+^* mice.

### Riccardin D decreases angiogenesis in intestinal polyps

**Figure 6:**
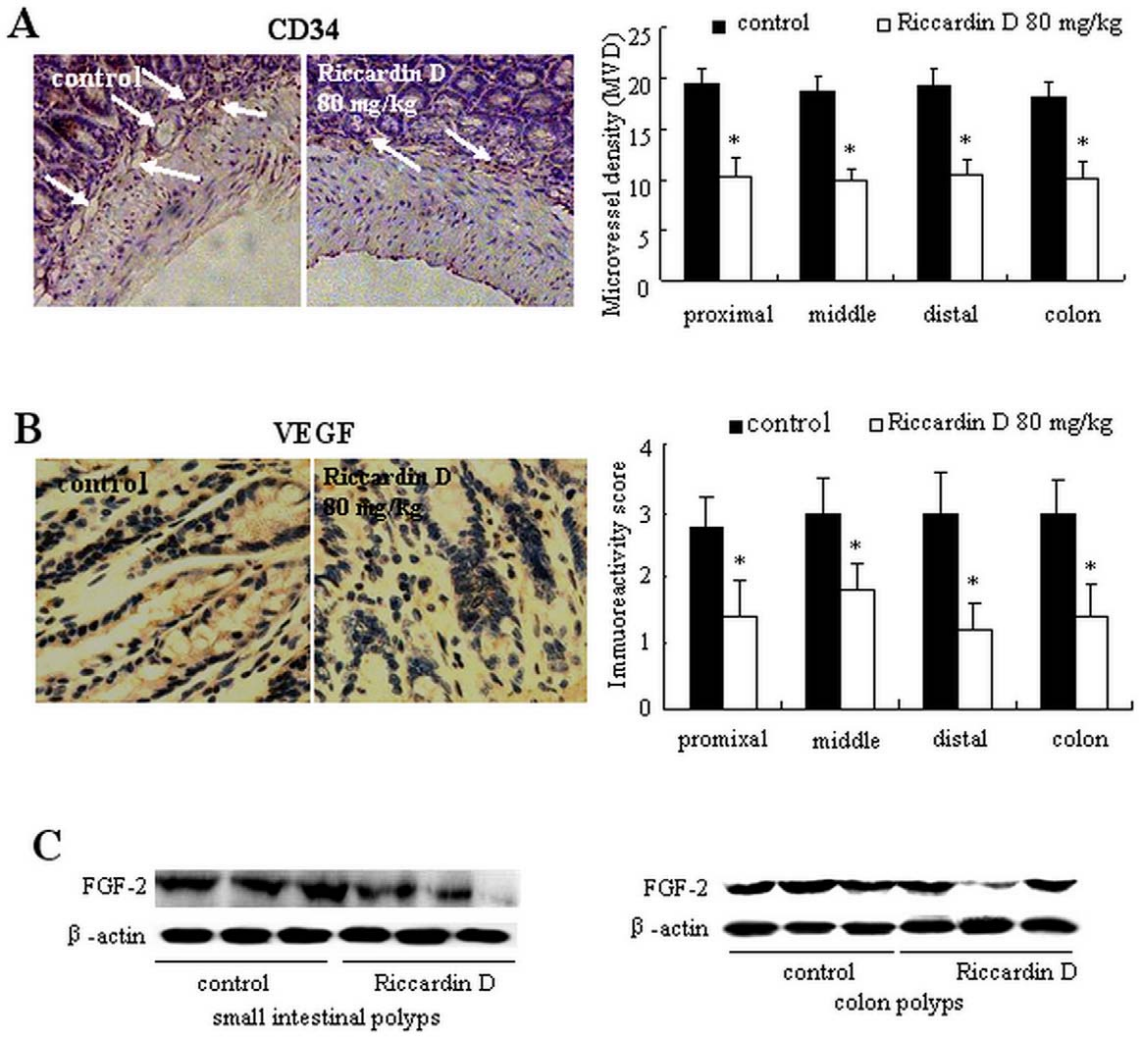
Riccardin D inhibited angiogenesis in intestinal polyps. (A): Immunohistochemical staining of CD34 for analysis of microvessel density (MVD) in polyps of 2–3 mm on small intestines and colons. MVD in polyps of different intestine segments was significantly inhibited by Riccardin D. (B): The expression of VEGF in polyps was inhibited by Riccardin D as estimated by immunohistochemical staining assay. The bars represent mean ± S.D. of six animals. *, p<0.001 *versus* control. (C): Riccardin D decreased the expression of FGF-2 in intestinal polyps as estimated by western blotting assay (n = 3).

**Figure 7:**
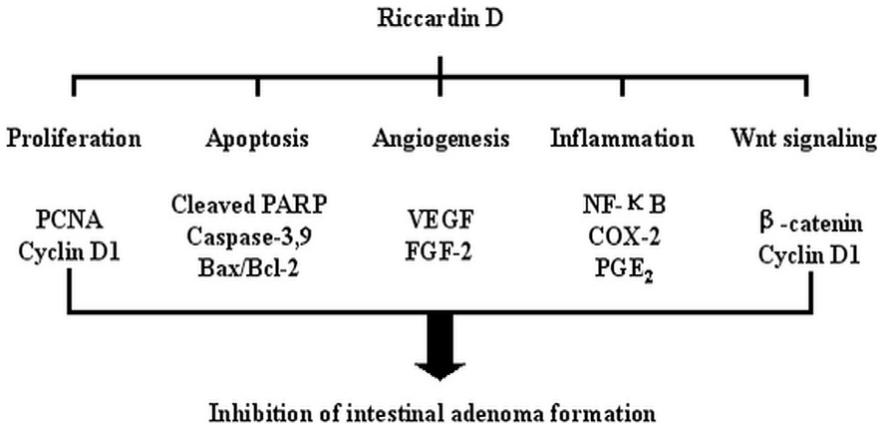
Summary for the pathways inhibited by Riccardin D in *APC^Min/+^* mice intestinal polyps.

We studied whether the inhibition of adenoma formation by Riccardin D was associated with its ability of antiangiogenesis. We first examined the microvessel density (MVD) in intestinal polyps of 2–3 mm size by CD34 immunohistochemical staining assay. The expressions of angiogenesis activators VEGF and FGF-2 in these intestinal polyps were then evaluated by western blotting analysis. As shown in Fig. 6A, the count of MVD in polyps from proximal, middle and distal part of intestinal and colon of Riccardin D-treated mice was significantly decreased by 49.2% (p<0.001), 47.0% (p<0.001), 48.6% (p<0.001), and 44.2% (p<0.001), respectively, compared with those from control mice. The examination of VEGF by immunohistochemistry showed that the intensity of immunoreactivity in intestinal polyps was significantly decreased in Riccardin D-treated mice (Fig. 6B). Quantitative data showed that Riccardin D decreases VEGF immunoreactivity scores in polyps by 57.1% (p<0.001) in proximal, 45.0% (p<0.001) in middle, 57.9% (p<0.001) in distal portions of small intestine, and 52.6% (p<0.001) in colon (Fig. 6B) of control. Fig. 7C showed the expression of FGF-2 in intestinal polyps as estimated by western blotting analysis. Quantitative data revealed that Riccardin D decreased FGF-2 expression by 53.4% (p<0.001) in small intestine and 61.8% (p<0.001) in colon of control. These results suggested that Riccardin D might target angiogenesis during the progression of intestinal adenoma formation. Importantly, the antiangiogenic effect of Riccardin D was specific and limited to polyps, because we did not observe considerable changes in the expression of these angiogenic factors in normal crypt-villus regions in intestine of control and Riccardin D-treated *APC^Min/+^* or wild-type C57BL/6 mice (data not shown).

## Discussion

This study demonstrated that administration of Riccardin D leads to the chemoprevention of intestinal adenoma formation in *APC^Min/+^* mice, a genetically predisposed animal model of human familial adenomatous polyposis (FAP) [25]. The key findings of this study include: (a) Riccardin D significantly reduced the number as well as the growth of intestinal polyps and prominently decreased the incidence of larger colonic polyps in *APC^Min/+^* mice; (b) the inhibitory effect of Riccardin D was associated with a decrease of growth and an increase of apoptosis in intestinal polyps; (c) Riccardin D decreased the chronic inflammation and modulated cytokines in intestinal polyps in favor of its inhibition of polyp growth; and (d) Riccardin D inhibited angiogenesis as evidenced by the decrease of MVD, VEGF and FGF-2 in intestinal polyps. Riccardin D did not show apparent toxicity to animals during the long-term treatment. These results suggested that Riccardin D could be a potential chemopreventive regimen for intestinal cancers derived from *APC* gene mutation. The proposed molecular pathways for the inhibitory effects of Riccardin D were summarized in Fig. 7.

In our previous studies, we had reported a group of macrocyclic bisbibenzyls, including Riccardin D, Plagiochin E and Marchantin C, which belong to the family of phenolic compounds [26]. These compounds possess a wide range of biological activities, such as anti-bacterial, anti-oxidation and cytotoxicity as well as inhibitory effects on cyclooxygenase, calmodulin and 5-lipoxygenase [27], [28]. Among them, Riccardin D has become the most promising therapeutic agent, for its high efficacy against human cancers with lower toxicity to animals. The structure-activity analysis suggested that the activity of these compounds might associate with the number and binding position of phenolic hydrogen [9]. We had studied the pharmacokinetics of Plagiochin E after oral (80 mg/kg) administration in rats [14]. The results showed a good bioavailability (10.6%) and a higher level of plasma concentration of Plagiochin E after observation (313.5 ng/ml) [14]. In this study, Riccardin D has the very similar chemical structure to Plagiochin E with three phenolic hydroxyl groups in double biphenyl bonds. We hypothesized that Riccardin D might possess the similar profile of pharmacokinetics given orally; thus, we dosed our animals with Riccardin D based on the pharmacokinetic calculation of Plagiochin E. The dose of Riccardin D used in this study showed high efficacy in the inhibition of intestinal adenoma formation in *APC^Min/+^* mice without obvious toxicity to animals. More pharmacokinetic and toxicity studies on Riccardin D are needed before it is being considered for clinical application.

The adenomatous polyposis coli multiple intestinal neoplasia (*APC^Min/+^*) mouse model represents the phenotypes of FAP, a hereditary CRC predisposition syndrome in humans [25], [29]. Patients with FAP develop multiple adenomas in the intestine, which eventually lead to the development of malignant adenocarcinomas through activation of the Wnt signaling pathway [6]–[8]. Cancer epidemic analysis showed that *APC* mutations were also found in approximately 80% of sporadic colorectal tumors. *APC* gene acts as a central gatekeeper protein in colorectal tumorigenesis [30]. Molecular studies suggested that the mutation of *APC* gene causes β-catenin to disassociate from cell membrane, and to migrate into nucleus. In the nucleus, β-catenin promotes the transcription of target genes that in turn leads to uncontrolled cell proliferation [31]. As a consequence, the cells affected will show a high expression of proliferation markers such as PCNA and cyclin D1 [6]–[8]. These biomarkers are most important downstream targets for the Wnt signaling pathway and have been previously shown to be highly expressed in the adenomas of *APC^Min/+^* mice [6]–[8]. Thus, the relevance of *APC^Min/+^* mouse model and the development of intestinal cancers in human were well recognized. In this study, Riccardin D was found to reduce the expressions of β-catenin, PCNA and cyclin D1 in intestinal adenoma, implying that the Wnt signaling pathway might be inhibited by Riccardin D.

Apoptosis, defined as programmed cell death, is an evolutionary conserved mechanism to balance cell proliferation essential for maintenance of tissue homeostasis. Tumor cells are characterized by uncontrolled cell proliferation without a balanced extent of apoptosis. Excessive adenoma growth and insufficient apoptosis are often associated with CRC development and progression, and the agents modulating them have immense potential in CRC chemoprevention or therapeutic intervention [32]. In this study, Riccardin D prevented adenoma growth and induced apoptosis of intestinal polyps by decreasing of PCNA and cyclin D1 in *APC^Min/+^* mice. Furthermore, Riccardin D induced the apoptosis of intestinal polyps by increasing of TUNEL-positive cells and activated caspase-dependent pathway. This apoptotic effect of Riccardin D in intestinal polyps might be part of the underlying mechanisms for the significant decrease in the number as well as the size of polyps in *APC^Min/+^* mice. Importantly, induction of apoptosis and inhibition of adenoma growth by Riccardin D were specific to adenomas without obvious effect on normal crypt-villus regions.

Enormous studies suggest that chronic inflammation plays an important role in intestinal tumorigenesis. Chronic inflammatory bowel disease accounts for about two thirds of the sporadic colorectal cancers [33]. Increase of inflammatory stress has been reported to be correlated with the development of intestinal polyposis in *APC^Min/+^* mice. Inactivation of *APC* gene in epithelial and stromal cells in human intestine and colon is associated with the NF-κB-COX-2 interaction [34]. In this interaction, the transcription factor kappa B (NF-κB), a proinflammatory transcription factor, acts as ‘first responder’ to various types of cellular stress such as free radicals, and pro-inflammatory cytokines such as TNF-α and bacterial components. NF-κB-p65, the active form of NF-κB, could stimulate the production of TNF-α and COX-2 [1], [2] and activate MMP-9, VEGF and cyclin D1 which have the NF-κB binding site in their transcription promoters [35]. Accumulation of NF-κB further increases the activation of COX-2 and the Wnt signaling pathway that is regulated by *APC* gene. COX-2, an immediate-early growth response gene product, is normally absent or expressed at very low levels in most cells but highly inducible in response to inflammatory cytokines, growth factors, and tumor promoters [1], [32]. One of the major arachidonic acid metabolites by COX-2 that is elevated in FAP patients is PGE_2_, implicating its major role in the formation of polyps on intestines [36]. PGE_2_ strongly stimulates the proliferation of intestinal polyps and suppresses apoptotic cell death of epithelial cells, leading to the expansion of polyps [37]. PGE_2_ also stimulates the polyps to expansion through increases of angiogenesis and provide a route for invasion and metastasis [38]. Therefore, NF-κB-COX-2 mediated inflammation displays the central roles in the stimulation of polyp growth on intestines. In this study, the NF-κB-COX-2 interaction was inhibited and its downstream product PGE_2_ was decreased by Riccardin D. Thus, the anti-inflammation mechanisms of Riccardin D likely played an important role in the observed suppression of polyps on intestines in *APC^Min/+^* mice.

Another important observation was that Riccardin D strikingly decreased the number of polyps that were larger in size (2–3 mm), suggesting that Riccardin D inhibited the progression of excessive proliferation of abnormal cells into polyps and also smaller polyps into larger ones. In intestinal polyps, angiogenesis is triggered when tumors grow beyond a minimal size (∼2 mm) [4], [15]. In this progression, VEGF and FGF-2 are important downstream targets of β-catenin that lead to endothelial cell proliferation to form vessels that support intestinal polyp growth and expansion [39], [40]. Riccardin D strongly inhibited the expressions of VEGF and FGF-2 and reduced the newly formed microvessel density in intestinal polyps. Therefore, the angiopreventive effect of Riccardin D could be an additional mechanism for its chemoprevention of intestinal adenoma formation.

In conclusion, our results showed antiproliferative, apoptotic, anti-inflammatory and anti-angiogenic effects of Riccardin D in intestinal polyps, which collectively contribute to its inhibition on spontaneous intestinal adenoma formation in *APC^Min/+^* mice. Our observation suggests that Riccardin D could be a promising regimen in chemoprevention against intestinal tumorigenesis.
